# Phase Diagram and Estimation of Flory-Huggins Parameter of Interaction of Silk Fibroin/Sodium Alginate Blends

**DOI:** 10.3389/fbioe.2020.00973

**Published:** 2020-08-18

**Authors:** Laise Maia Lopes, Mariana Agostini de Moraes, Marisa Masumi Beppu

**Affiliations:** ^1^School of Chemical Engineering, University of Campinas, Campinas, Brazil; ^2^Department of Chemical Engineering, Federal University of São Paulo, Diadema, Brazil

**Keywords:** silk fibroin, sodium alginate, phase separation, blends, thermodynamic, biomaterials

## Abstract

Silk fibroin (SF) and sodium alginate (SA) are natural polymers used to produce biomaterials. One of the strategies to improve the properties of these products is to prepare blends with them, which are partially miscible. Phase separation is observed, therefore, the thermodynamic analysis of this system is important to predict the final state and composition of this blends. This study explored blends with a different initial composition of SF, SA, and water (WA) at 25°C and neutral pH. After phase separation, two phases were identified, one rich in SF and other rich in SA. The Flory-Huggins parameters of interaction of polymer-solvent and polymer-polymer were estimated using the extended equation and data of phase equilibrium, their values indicates the partial miscibility of the polymers.

## Introduction

Blends of Silk fibroin (SF) and sodium alginate (SA) have been studied since the beginning of the 90’s years (Liang and Hirabayashi, 1992). Most of the works with these two polymers explore the potential of the blends as a biomaterial due to their characteristics such as biocompatibility, biodegradability and low toxicity (de Moraes and Beppu, 2013; de Moraes et al., 2014; Ming and Zuo, 2014; Zhang et al., 2015; Wang et al., 2016). Despite that, the commercialization of these products is not a reality due to the complexity of the system formed by these polymers.

Silk fibroin is a protein produced by arthropods like spiders, mites, bees and silkworms, the last one is more used due to the facility of domestication (Kundu et al., 2013), the main producer worldwide is *Bombyx mori* silkworm (Koh et al., 2015). It has good mechanical properties with high tensile strength and high thermal resistance with a temperature of degradation close to 300°C (Kundu et al., 2013; Koh et al., 2015). SF has been explored to develop new materials such as porous materials, scaffolds, wound dressings, hydrogels, nano-particles and as a substrate in optics and sensors (Kim et al., 2005; Nogueira et al., 2011; Wenk et al., 2011; Calamak et al., 2014; Li et al., 2017; Wang et al., 2020).

One of the challenges of working with SF is its metastable solution behavior. The transition of α-helix conformation to β-sheet occurs spontaneously and can speed up by temperature, mechanical treatments and changes in solvent and salt concentration (Sohn et al., 2004; Matsumoto et al., 2006). This transition in SF chain conformation is widely studied in literature because it can clarify the self-assembly mechanism and nanofibrils formation of SF (Lu et al., 2012; Bai et al., 2013; Zhong et al., 2015; Li et al., 2018). This information is important for designing new materials. It is reported that it depends on thermodynamic and, also, kinetic variables (Lu et al., 2012; Bai et al., 2013).

Another important parameter to be investigated is the behavior of SF in aqueous solution. SF is a non-water soluble protein, to obtain its solution it is necessary to use a saline solution, usually containing LiBr or CaCl_2_. The dialysis process could influence the SF conformation by changing the osmotic pressure, the temperature and the salt concentration after dialysis (Sohn et al., 2004; Nogueira et al., 2011; Yang et al., 2013; Ribeiro et al., 2014). The knowledge about how SF conformation can be adjusted in SF solution is fundamental since it could influence the self-assembly process and consequently the properties of new materials.

Sodium alginate is a polysaccharide extracted from brown seaweeds and some bacteria (Agulhon et al., 2014). It is a copolymer formed by the α-L guluronic acid (block G) and β-D mannuronic acid (block M), their fraction and sequence in SA chain depend on the species of brown seaweeds that SA was extracted and can influence in the properties of the products developed (Draget et al., 1997). It is a natural polymer very explored in the development of different products, from the food industry, as an emulsifier, to the biomaterials field due to its facility to form films and hydrogels. Some SA applications are as cell encapsulation, wound dressing and drug delivery (Meng et al., 2010; Brachkova et al., 2011; Feng et al., 2014; Leung et al., 2014; Bhutani et al., 2015; Sarker et al., 2015).

Due to its use in the food industry, some studies investigated the phase separation of systems composed by alginate and other proteins, such as gelatin, casein and pea protein (Antonov et al., 1996; Panouillé and Larreta-Garde, 2009; Antonov and Moldenaers, 2011; Mession et al., 2012). Although SF and those proteins have different characteristics, the results could be useful to understand the SF-SA system. In those studies, phase separation is observed, as well as the presence of globular structures even in low concentration of polymers (Antonov and Moldenaers, 2011; Mession et al., 2012). These structures are also observed by scanning electron microscopy in our previous study of SF-SA blends (de Moraes et al., 2014; Lopes et al., 2018), which investigated the physical-chemical behavior of the blends.

Protein-polysaccharide systems are complex and in many cases, phase separation is observed (Pacek et al., 2000; Mession et al., 2012). Miscible blends are rare, being considered almost an exception in systems with macromolecules (Utracki and Manias, 2014). A miscible blend must attempt two conditions, Eqs 1 and 2:

(1) $$\Delta \,G_{m}=\Delta \,H_{m}-T\,\Delta \,S_{m}<0$$

(2) $${\left(\frac{\partial^{2}\Delta \,G_{m}}{\partial \varphi_{i}^{2}}\right)}_{T,P}>0$$

where Δ*G*_*m*_ is the Gibbs free energy of mixing, Δ*H*_*m*_ is the enthalpy of mixing, T is the temperature, Δ*S*_*m*_ is the entropy of mixing, ϕ_*i*_ is the volumetric fraction.

For polymers, the entropic contribution is small due to the low probability of molecular rearrangement (Mishra et al., 2017). Thus, the enthalpic contribution plays an important role in polymeric systems. So, macromolecules that interact through strong bonds, for example, hydrogen bonds, can form partially miscible systems. The interaction between SF and natural polysaccharides is well established in the literature and it can occur by hydrogen bonds and covalent bonds (de Moraes et al., 2014; Zhang et al., 2015; Wang et al., 2019). In protein-polysaccharide aqueous systems, intermolecular complexes are formed and macroscopically phase separation occurs by thermodynamic incompatibility (Mession et al., 2012). In a recent study, we focused on understanding the mechanism of phase separation of SF-SA system and the physical-chemical characteristics of those blends (Lopes et al., 2018). The literature presents just a few studies about the application of SF-SA blends and there is a lack of literature about the phase behavior of these blends.

In the present study, the phase separation and equilibrium of the ternary system composed by SF, SA, and water (WA) was experimentally investigated at 25°C and neutral pH. The quantification of each phase was made using different methods. The identification of the region rich in SF or SA is necessary to predict the behavior of blends with different initial conditions that will influence the final state of the blend and consequently the process and final product.

## Materials and Methods

### Preparation of Solutions: Silk Fibroin and Sodium Alginate

Silk fibroin solution was prepared following the method previously published (de Moraes et al., 2014). Briefly, *Bombyx mori* silkworm cocoons (Bratac, Brazil) were degummed using 1 g.L^–1^ Na_2_CO_3_ solution in a thermostatic bath at 85°C (Nova Analitica, Brazil) for 30 min, the process was repeated other two times. The fibers obtained were washed with deionized water and maintained at room temperature until completely dry. They were milled using a sieve with 10 mesh of diameter and then solubilized in a solution of CaCl_2_: Ethanol: water (1:2:8 molar ratio). To remove the salts, the solution was dialyzed using a dialysis membrane (MWCO 3.5 kDa, Thermo Fisher Scientific, EUA) in ultrapure water (1:10 ratio). To remove the insoluble parts that are formed during dialysis, the solution was centrifuged for 30 min and 2300 relative centrifugal force (RCF). The final concentration was calculated by gravimetry and was around 4% (w/v).

Sodium alginate (Sigma-Aldrich, United States) extracted from *Macrocystis pyrifera* were dissolved in deionized water with a concentration of 2% (w/v). The solution was kept 3 days, so the water could solvate the powder, then the solution was homogenized by stirring.

### Blend Preparation

The SF and SA blends were prepared in seven different mass proportions, as shown in Table 1. These compositions were chosen following previous studies (Lopes et al., 2018) which showed that for more concentrated systems, i.e., with less water, hydrogel formation would occur. Thus, it would not be possible to make a quantitative analysis of each phase. To prepare the blends, SF was slowly added to SA solution under stirring, the system was kept in a thermostatic bath at 25°C (Nova Analitica, Brazil). After 8 days, each phase was collected and the components were quantified. The notation used to refer the blends was SA_*x*__%_/SF_*y*__%_/WA_*z*__%_ where x, y, and z are the mass fraction of each component in the blend.

**TABLE 1:** Initial mass fraction (%) of each component in the SF and SA blend.

Blend	SF	SA	WA
SF_0_._5_/SA_0_._5_/WA_99_	0.5	0.5	99
SF_1_._5_/SA_0_._5_/WA_98_	1.5	0.5	98
SF_1_._0_/SA_1_._0_/WA_98_	1.0	1.0	98
SF_0_._5_/SA_1_._5_/WA_98_	0.5	1.5	98
SF_1_._5_/SA_1_._5_/WA_97_	1.5	1.5	97
SF_1_._0_/SA_2_._0_/WA_97_	1.0	2.0	97
SF_0_._5_/SA_2_._5_/WA_97_	0.5	2.5	97

### Zeta Potential

Zeta potential was performed using Malvern Zetasizer Nano ZS (Malvern, United Kingdom). It was measured using a zeta universal dip cell and polystyrene cuvettes. The polymers concentration was 1 g.L^–1^ and the samples were measured three times. The values of the refractive index and absorbance used were 1.33 and 0.001 a.u for SA and 1.45 and 0.001 a.u for SF. The absorbance was measured using the Varioskan LUX multimode microplate reader (Thermo Fisher Scientific, United States) and the refractive index was measured using a refractometer (Instrutherm, Brazil). The pH of SF and SA solution was 6.77 and 7.63, respectively. Zeta potential was performed to analyze the blends by mixing the SF and SA solution with the same concentration in different volume proportions, 75–25, 50–50 and 75–25 (SA-SF).

### Optical Microscopy

The optical microscopy was used to analyze the morphology of microparticles formed after blend preparation. The optical microscopy was performed using the E200 optical microscope (Nikon, Japan). The image was analyzed using ImageJ software.

### Phase Quantification

**FIGURE 1:**
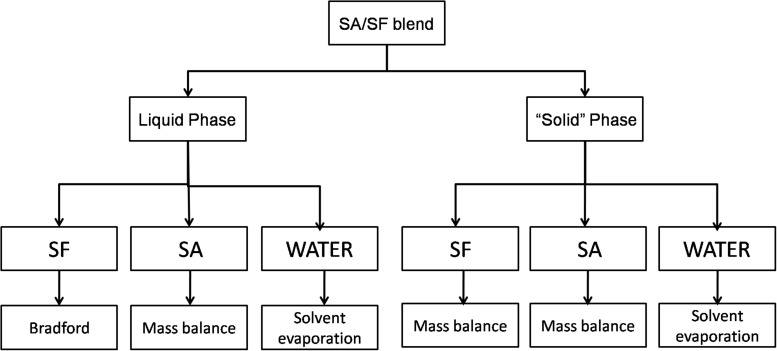
Scheme showing the methodology used to quantify the different substances in liquid and solid-like phase.

After phase separation, the blends were centrifuged for 15 min and 2300 RCF, the final state of SA/SF blends is a liquid phase and a “solid” one that looks like a hydrogel and it is mostly composed by SF with water trapped in the structure. The analysis used to quantify each phase is described in Figure 1.

Water was quantified gravimetrically by solvent evaporation in the liquid and solid phase in an oven until the weight was constant. SF in the liquid phase was quantified by Bradford assay (Bradford, 1976), a consolidated method to quantify proteins using the Coomassie Blue G-250 stain with maximum absorbance in the range between 465 and 595 nm. This method was chosen because carbohydrates do not interfere with the results. It was prepared a calibration curve from 0 to 100 μg/mL using Bradford reagent (Amresco, United States) that was analyzed at 590 nm using a 24 plate-well in a spectrophotometer (Tecan M200 Pro, Switzerland). In the solid-like phase, it was not possible to use the same method because SF was insoluble, so it was quantified by mass balance using Eq. 3.

(3) $$m_{s\,f\,s\,p}=m_{m\,s\,p}-m_{w\,a\,s\,p}-m_{m\,a\,s\,p}$$

where m_*msp*_ is the total mass of solid phase, m_*sfsp*_ is the SF mass in the solid-like phase, m_*wa*__*s*__*p*_ is the water mass in the solid-like phase and m_*sa*__*s*__*p*_ is the SA mass in the solid-like phase.

The SA was quantified by mass balance in both phases using Eqs 4 and 5.

(4) $$m_{s\,a\,l\,p}=m_{m\,l\,p}-m_{s\,f\,l\,p}-m_{w\,a\,l\,p}$$

where m_*sal*__*p*_ is the SA mass in the liquid phase, m_*mlp*_ is the total mass of liquid phase, m_*sfl*__*p*_ is the SF mass in the liquid phase (determinate by Bradford assay) and m_*wal*__*p*_ is the water mass in the liquid phase.

(5) $$m_{s\,a\,s\,p}=\left(m_{t}*a\right)-m_{s\,a\,l\,p}$$

where m_*sas*__*p*_ is the SA mass in the solid phase, m_*t*_ is the blend mass, α is the SA mass fraction in the blend, and m_*sal*__*p*_ is the SA mass in the liquid phase.

### Flory-Huggins Parameter of Interaction

The Flory-Huggins model is a classic mathematical approach for polymer systems. It uses the balance between the entropic and enthalpic contributions (Eqs 6 and 7), regarding the size and form effect and the intermolecular interactions.

(6) $$\frac{\Delta \,S}{R}=\Sigma x_{i}\ln\varphi {}_{i}$$

(7) Δ⁢HR⁢T=(∑xii⁢ViVs)⁢∑∑iχi⁢jTj>i⁢φi⁢φj

where R is the gas constant, T is the temperature, x_*i*_ is the molar fraction of component i, V_*i*_ e V_*s*_ are the volume per amount of matter of pure component i and for solvent, respectively; χ_*ij*_ is the Flory-Huggins parameter of interaction for component i and j, φ_*i*_ and φ_*j*_ are the volumetric fractions of component i and j.

By replacing Eqs 6 and 7 in Eq. 1, the extended expression of the Flory-Huggins equation is obtained, which is usually simplified for use in a polymer-solvent system. The Flory-Huggins parameter can be, also, calculated using the activity value of each component in each phase. This is useful because in the equilibrium the chemical potential of each component in each phase is equal, and using the data of equilibrium is possible to calculated Flory-Huggins parameter solving a non-linear equation system, described by Eqs 8–10 for a ternary system (Favre et al., 1996).

(8) $$l\,n\,a_{1}=\ln\varphi_{1}+\left(1-\varphi_{1}\right)-\left(\frac{V_{1}}{V_{2}}\right)\,\varphi_{2}-\left(\frac{V_{1}}{V_{3}}\right)\,\varphi_{3}+\left(\left(\chi_{12}\,\varphi_{2}+\chi_{13}\,\varphi_{3}\right)\,\left(\varphi_{2}+\varphi_{3}\right)\right)-\chi_{23}\,\left(\frac{V_{1}}{V_{2}}\right)\,\varphi_{2}\,\varphi_{3}$$

(9) $$l\,n\,a_{2}=\ln\varphi_{2}+\left(1-\varphi_{2}\right)-\left(\frac{V_{2}}{V_{1}}\right)\,\varphi_{1}-\left(\frac{V_{2}}{V_{3}}\right)\,\varphi_{3}+\left(\left(\chi_{12}\,\varphi_{1}\,\left(\frac{V_{2}}{V_{1}}\right)+\chi_{23}\,\varphi_{3}\right)\,\left(\varphi_{1}+\varphi_{3}\right)\right)-\chi_{13}\,\left(\frac{V_{2}}{V_{1}}\right)\,\varphi_{1}\,\varphi_{3}$$

(10) φ+1φ+2φ=31

where *a*_*1*_ and *a*_*2*_ are the activity of component 1 and 2, respectively; V_1_, V_2_ and V_3_ are the volume per amount of matter of component 1, 2, and 3, respectively; χ_*12*_, χ_*13*_, and χ_*23*_ are the Flory-Huggins parameter of component 1–2, 1–3, and 2–3, respectively.

The Flory-Huggins model is used to describe various polymeric systems, due to its simplicity and the possibility of its interaction parameter being determined experimentally by calorimetric techniques or, osmotic pressure, for example (Safronov et al., 2009; Kim et al., 2010). The extended equation can be used as an initial model to describe and understand the forces involved in polymer-polymer and polymer-solvent interactions.

## Results

### Physical Aspects of the SA/SF Blends

**FIGURE 2:**
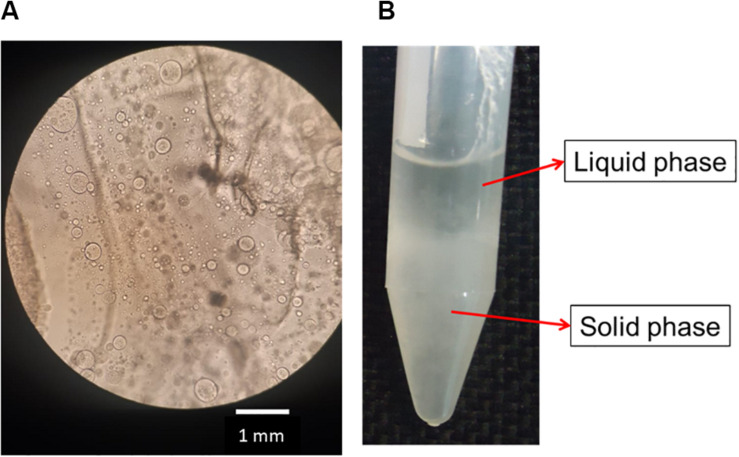
Image of optical microscopy of silk fibroin and sodium alginate blend after preparing **(A)**. Photo of the SA_0_._5_/SF_0_._5_/WA_99_ blend showing phase separation and formation of a liquid and solid-like phase **(B)**.

**FIGURE 3:**
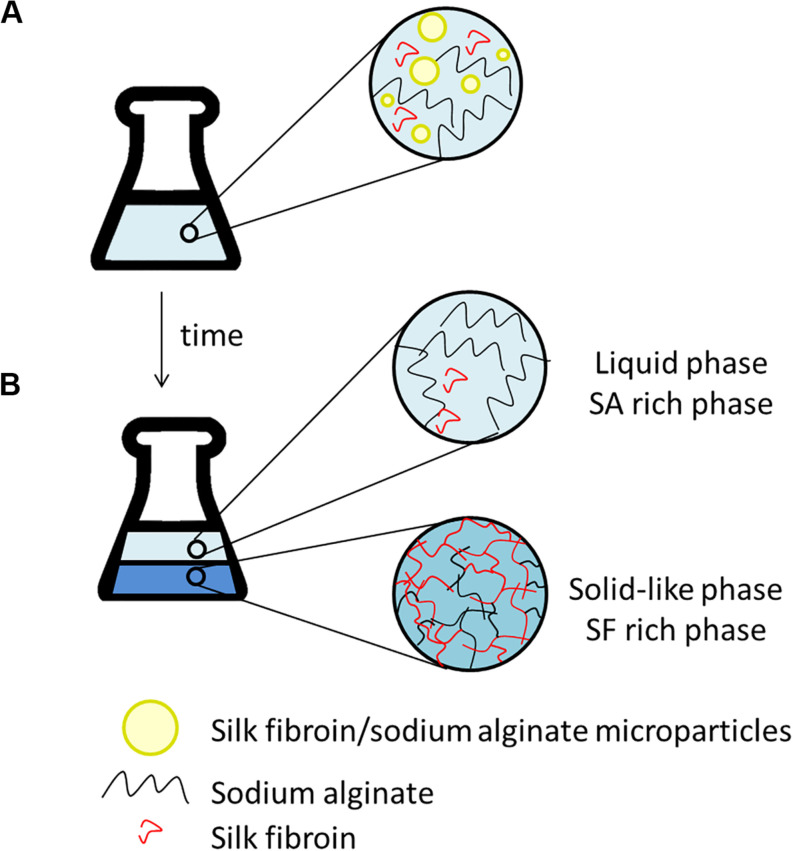
Scheme illustrating the time evolution of phase separation that could be occurring in the silk fibroin/sodium alginate blends. **(A)** After preparing the blends: microparticles formation (observed in microscope). **(B)** After some days: liquid phase enriched with SA, coalescence of globules and hydrogel (solid-like phase) formation. The water is present in both stages **(A,B)** and represented by the blue background.

After the addition of silk fibroin in sodium alginate solution, for blend preparation, it was possible to notice that the solution became cloudy immediately, even in blends with low polymer concentration, for instance, SF_0_._5_SA_0_._5_WA_99_. When observing the blend in optical microscopy, microstructures with a diameter of 0.247 ± 0.120 mm were detected (Figure 2A). The formation of these structures, like drops, was also verified in other protein-polysaccharide systems, such as alginate-casein and alginate-pea protein (Pacek et al., 2000; Antonov and Moldenaers, 2011; Mession et al., 2012). After 1 day, it was possible to notice two phases, shown in Figure 2B. The proportion of each phase changes over the days, the solid-like phase becomes denser, indicating that even if phase separation started after 1 day (macroscopically), the system was not in thermodynamic equilibrium. The evolution of this phenomenon was investigated by static light scattering (Lopes et al., 2018) and the system was considered in equilibrium when changes in the scattering profile were not observed anymore, which happened after the eighth day. It is possible that over time, the microstructures observed in the microscope, collapse and precipitate forming the solid-like phase (Figure 3).

The hydrogel formation, here called the solid-like phase, is a spontaneous phenomenon. It could happen due to the hydrophobic interaction and intra and intermolecular hydrogen bonds, changing the SF conformation from α-helix to β-sheet, precipitating the protein and turning it stable in water (Matsumoto et al., 2006). Some parameters can contribute to the hydrogel formation, such as protein concentration, decreasing pH, chemical crosslinking and temperature (Nogueira et al., 2011; Yin et al., 2017). In previous studies, it was observed that the increase in temperature could accelerate the hydrogel formation from SF solution, as well as, the presence of poly(ethylene oxide) (PEO), a water-soluble polymer (Kim et al., 2004; Nogueira et al., 2011; Zhong et al., 2015; Yin et al., 2017). Based on the literature, SA may have the same influence, accelerating the formation of the solid-like phase.

### Zeta Potential

Table 2 shows the values of zeta potential. that was negative for both polymers. For SF this parameter is close to zero, showing a balance of charges between the amino and carboxyl groups. The pI for SF is around 4, so in neutral pH, there is a predominance of negative charges, which justify the value obtained (Wenk et al., 2011). This zeta potential value also indicates that the SF solution is unstable, based on DLVO theory (Derjaguin and Landau, 1941), which could explains the spontaneous hydrogel formation after some days. The SA zeta potential value was −57.9 mV. At neutral pH, the carboxyl groups are deprotonated (pKa = 3.4–3.6) (Draget et al., 1997), so the polymer chain will be negatively charged.

**TABLE 2:** Values of zeta potential (mV) of SF, SA, and blends. Solution concentration for both polymers was 1 g/L and solution pH was 6.77 for SF and 7.63 for SA.

	Zeta potential (mV)
SF	−12.7 ± 0.3
SA	−57.9 ± 0.4
SA_75_/SF_25_	−64.0 ± 2.8
SA_50_/SF_50_	−50.0 ± 2.3
SA_25_/SF_75_	−29.4 ± 0.5

Analyzing the zeta potential values of each polymer is reasonable to conclude that there will be an electrostatic repulsion between the polymers. At the same time, there will be also an interaction between the amino groups from SF and the carboxyl groups of SA. The results of blends zeta potential show that for the ones with more SF in the composition, the zeta potential is higher showing charge compensation phenomenon.

### Phase Quantification and Estimation of Flory-Huggins Parameter of Interaction

**FIGURE 4:**
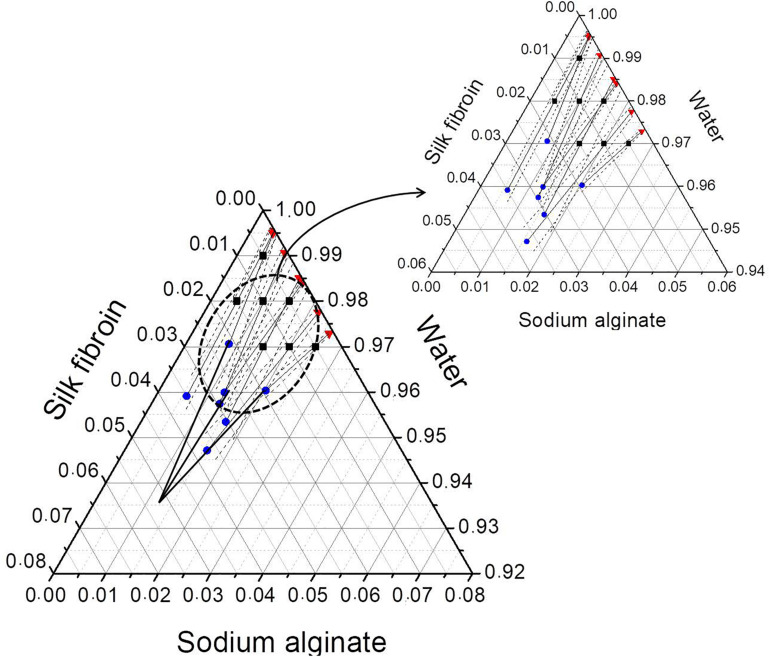
Ternary phase diagram of SF, SA, and water at 25°C showing the conversion of tie lines to the “true precipitate” point and the region of variability in the zoom detail. Feed (■), SF-rich phase in red (▲), SA rich-phase in blue (🌑) connected by tie lines (—) and region of variability (—⋅—). All the concentrations are given in mass fraction.

After phase separation, a phase rich in SA (liquid) and another rich in SF (solid-like) are observed, the experimental results for the composition of each phase are shown in Table 3 and plotted in the ternary graph shown at Figure 4. Water is the most predominant component of the phases, even in the solid-like one, where water is probably entrapped between the polymer chains, and then the hydrogel formation occurs, as proposed in Figure 3. The tie lines are very close to each other and it was observed that each blend presents some variability, that is expected due to the characteristics of SF and SA solutions, which form a dispersion and may have a small variation in the same sample. Besides, there is inherent variability in the measurements, therefore, the establishment of this region is important.

**TABLE 3:** Average mass fraction (%) of feed, SF rich phase (solid) and SA rich phase (liquid) at 25°C, *n* = 3.

Mass fraction (%) of each component in feed			Mass fraction (%) of SF rich phase (solid)			Mass fraction (%) of SA rich phase (liquid)		
SF	SA	WA	SF	SA	WA	SF	SA	WA
0.5	0.5	99	2.12 ± 0.47	0.82 ± 0.39	97.06 ± 0.08	0.01 ± 0.01	0.44 ± 0.08	99.55 ± 0.08
1.5	0.5	98	3.50 ± 0.23	0.59 ± 0.14	95.91 ± 0.09	0.06 ± 0.02	0.44 ± 0.11	99.50 ± 0.11
1.0	1.0	98	2.96 ± 0.10	1.30 ± 0.23	95.74 ± 0.32	0.05 ± 0.03	0.88 ± 0.10	99.07 ± 0.10
0.5	1.5	98	2.74 ± 0.33	1.27 ± 0.13	95.99 ± 0.04	0.04 ± 0.03	1.55 ± 0.03	98.41 ± 0.03
1.5	1.5	97	3.04 ± 0.12	1.62 ± 0.36	95.34 ± 0.52	0.05 ± 0.02	1.44 ± 0.29	98.50 ± 0.29
1.0	2.0	97	3.71 ± 0.03	1.58 ± 0.26	94.71 ± 0.33	0.07 ± 0.02	2.18 ± 0.08	97.75 ± 0.08
0.5	2.5	97	1.93 ± 0.22	2.04 ± 0.05	96.03 ± 0.18	0.09 ± 0.01	2.62 ± 0.03	97.29 ± 0.03
								

The phase diagram can be analyzed by the method presented by Popova et al. (2008) that is based on two major principles. The first one is that the protein-rich phase is not a single phase but a mixture of two: a supernatant and a “true” precipitate, which cannot be separated. The other is that the extension of the tie lines would converge to one point, which represents the “true” precipitate that in this case would be composed mostly by SF. The phase diagram shows that the tie-lines converge to a point with a high concentration of SF (5.4 wt% of SF, 1.25 wt% of SA, and 93.35 wt% of water). In practice, the “true” precipitate could not be obtained, because, at this concentration, the system would be in the “hydrogel” zone, therefore, it would not be possible to observe two distinguished phases.

**FIGURE 5:**
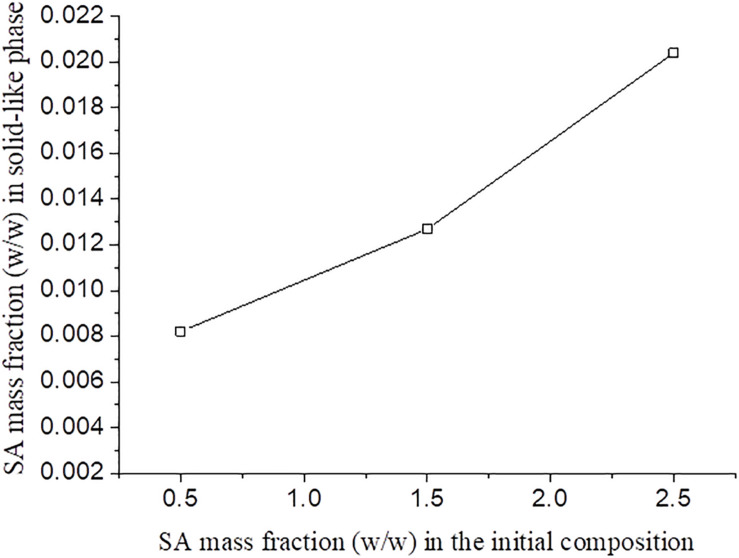
SA mass fraction in the solid-like phase after phase separation for different SF initial concentration as function of the SA initial concentration.

It was observed that, for blends with the same SF initial concentration, the increase of SA initial concentration resulted in a higher concentration of this polymer in solid-like phase (Figure 5). It was expected that an increase in the polysaccharide concentration would lead to a more concentrated SF solid-like phase since the SA has more affinity to the solvent and would stay in solution. Thus, probably part of the SA rich phase could be entrapped within the SF rich phase, as observed in Figure 5, as a consequence of SA viscosity (Mession et al., 2012). This behavior is observed in other protein-polysaccharide systems, such as alginate-pea protein and soy protein-κ- carrageenan (Li et al., 2008; Mession et al., 2012).

**FIGURE 6:**
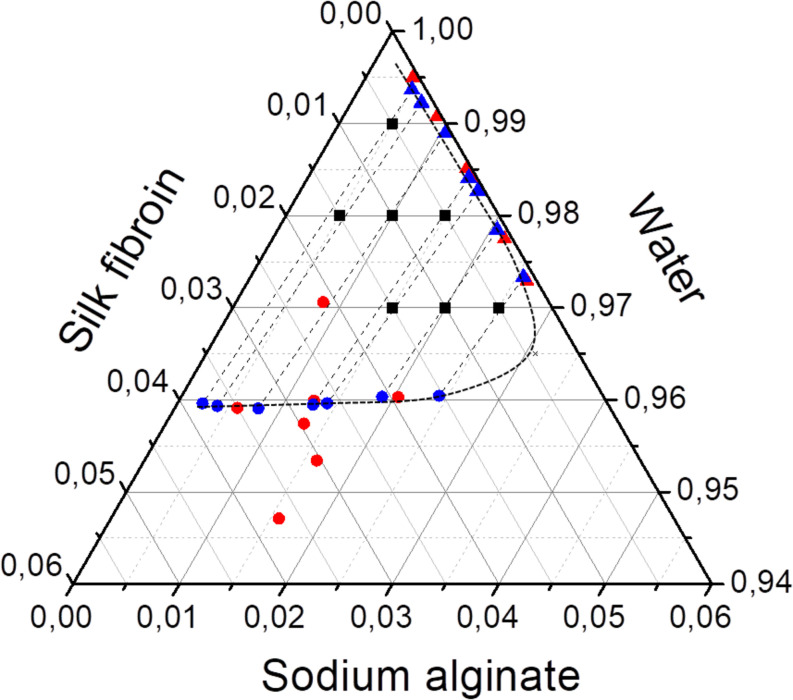
Ternary phase diagram of SF, SA, and water at 25°C with the feed composition (■), experimental (red), and calculated data (blue) of liquid (▲) and solid-like phase (🌑). Binodal curve plotted with calculated data (– –).

The three Flory-Huggins parameters of interaction were estimated by solving the non-linear equation system described by Eqs 8–10, the values are reported in Table 4. Using those values and the equilibrium data, new mass fractions were calculated by fixing the composition of two values and using Eq. 8 for both phases. The theoretical mass fractions for each component in liquid and solid-like phases are shown in Table 5, and the ternary phase diagram with the calculated and experimental data is shown in Figure 6. The binodal curve was plotted using the Flory-Huggins model. It was not possible to obtain the experimental binodal curve, because this is, usually, obtained by cloudy point experiments. As previously described, when mixing the silk fibroin solution in sodium alginate solution, the formation of microstructures (like drops) is observed, even in blend with low polymer concentration, which makes it difficult to determinate the cloudy point.

**TABLE 4:** Values of Flory-Huggins parameters of interaction (χ_*SAWA*_, χ_*SFWA*_, and χ_*SFSA*_) for silk fibroin-sodium alginate-water system at 25°C.

χ_*SAWA*_	χ_*SFWA*_	χ_*SFSA*_
−0.004	0.015	−0.424

**TABLE 5:** Mass fraction of silk fibroin, sodium alginate, and water calculated using the parameter of Flory-Huggins estimated by the non-linear system of Eqs. 8, 9, and 10.

Mass fraction (%) of each component in feed			Mass fraction (%) of SF rich phase (solid)			Mass fraction (%) of SA rich phase (liquid)		
SF	SA	WA	SF	SA	WA	SF	SA	WA
0.5	0.5	99	3.80	0.24	95.96	0.13	0.50	99.37
1.5	0.5	98	3.68	0.39	95.93	0.11	0.66	99.23
1.0	1.0	98	3.31	0.79	95.90	0.05	1.05	98.90
0.5	1.5	98	2.77	1.28	95.95	0.08	1.51	98.41
1.5	1.5	97	2.63	1.41	95.96	0.07	1.67	98.27
1.0	2.0	97	2.08	1.89	96.03	0.09	2.06	97.84
0.5	2.5	97	1.54	2.42	96.04	0.10	2.57	97.33

## Discussion

The system formed by SF, SA, and water is complex and partially miscible. That is a common phenomenon observed in protein-polysaccharide systems. A study conducted by Grinberg and Tolstoguzov (1997) showed almost a hundred different systems considered thermodynamically incompatible. After blend preparation, it was observed the formation of microstructures, like drops, in solution, indicating that phase separation occurs almost instantly. After some days, the polymers formed two phases, one liquid and rich in SA, another solid-like and rich in SF. It was observed that the kinetics of phase separation is slow; it takes 8 days to the system to reach equilibrium. The zeta potential showed that at the pH used for blends preparation, SF and SA are negatively charged, which favors the electrostatic repulsion. Despite that, the chemical interaction is observed, with a charge compensation between the polymers, because the SF has groups positively charged, NH^+^_3_, and SA has groups negatively charged, COO^–^, which could interact through hydrogen bonds (Shang et al., 2013). The system with more quantity of SF presented zeta potential closer to zero, due to the interaction between carboxyl and amino groups.

In systems containing biopolymers, two different types of phase separation can be observed: associative phase separation or segregated phase separation, it depends on the interactions between the biopolymer and the solvent (Doublier et al., 2000). The first one occurs when the polymer has more affinity to the solvent than to the other polymer; generally, the Flory-Huggins parameter is positive. The second one occurs when the polymer-polymer interactions are favored, and normally the Flory-Huggins parameter is negative (Doublier et al., 2000). It is observed the formation of complexes or polymer coacervate. The experimental results for the system studied and the interpretation from the point of view of phase equilibrium thermodynamics showed that the solid-like phase seems to be a mixture between SF and a supernatant formed by alginate water that coacervates, forming a hydrogel. The formation of a “true precipitate” could not be possible in this system because it requires a high amount of SF, which in practice leads to hydrogel formation. Due to the characteristics of the system, the formation of a coacervate and negative Flory-Huggins parameter of interaction, probably the type of phase separation observed in silk-alginate system is the segregated one. Another fact that corroborates is that segregated phase separation usually occurs when anionic polysaccharide is mixed with proteins with a negative charge (close to neutrality), which is the case of silk-alginate blends.

The value of the Flory-Huggins parameter of SF-SA interaction is negative, while SA-water and SF-water parameters of interaction are negative and positive, respectively. If we analyze the Equation 7 the Flory-Huggins parameter is related to the enthalpic contribution, which for polymers is more significant than the entropic contribution since the polymers chains have low mobility and fewer possibilities of molecular configuration. Generally, the enthalpic term is also unfavorable to the establishment of a miscibility system, mainly for polymers that interact through van der Walls bond (Kim et al., 2010). However, for polymers that interact by ionic bonds, dipole-dipole, or hydrogen bonds, miscibility can be achieved. Therefore, if the polymers have a good chemical interaction, represented by the negative F-H parameter, it is reasonable to expect a miscible or semi-miscible blend, which occurs in the SF-SA blend. The polymer-polymer parameter is very important, but the phase separation phenomenon takes into account the polymer-solvent interaction, and depending on this balance of forces, phase separation will be favored or not. That is why it is important to use the F-H extended equation, so this analysis can be done more accurately.

The Flory-Huggins model is widely used due to the facility to obtain experimentally the parameter of interaction. It is based on three hypotheses (Schmid, 2011):

1. The polymer conformation is considered an ideal chain, independent on the composition;
2. The polymers are considered incompressible and the monomers occupy equal volumes;
3. The local composition fluctuations are neglected.

In practice, none of them is valid, since the polymer conformation depends on composition, the monomers volumes are not equal and the composition fluctuation has an important influence in phase separation. These variations can have a significant influence on the real Flory-Huggins parameter, especially for natural polymers that already have intrinsic variability in their composition. Despite the limitations, the model presented an acceptable correlation, mainly in the liquid phase, and can give important information about the studied system. It can be used for initial studies and its physical-chemical interpretation must take into account all the limitations.

The understanding of a complex system, such as those formed by protein and polysaccharides, is important because they have been used extensively in our daily life. Therefore, fundamental studies are important, to predict and understand the system behavior, allowing the products made by these polymers to be produced and available in the market.

## Data Availability Statement

The datasets generated for this study are available on request to the corresponding author.

## Author Contributions

MB conceived of the presented idea. LL performed the experiments and analyzed the data. MM and MB verified the analytical methods. and supervised the findings of this work. All authors discussed the results and contributed to the final manuscript.

## Conflict of Interest

The authors declare that the research was conducted in the absence of any commercial or financial relationships that could be construed as a potential conflict of interest.

## References

[B1] Agulhon P. Robitzer M. Habas J.-P. Quignard F. (2014). Influence of both cation and alginate nature on the rheological behavior of transition metal alginate gels. *Carbohydr. Polym.* 112 525–531. 10.1016/j.carbpol.2014.05.097 25129777

[B2] Antonov Y. A. Lashko N. P. Glotova Y. K. Malovikova A. Markovich O. (1996). Effect of the structural features of pectins and alginates on their thermodynamic compatibility with gelatin in aqueous media. *Food Hydrocoll.* 10 1–9. 10.1016/S0268-005X(96)80047-6

[B3] Antonov Y. A. Moldenaers P. (2011). Structure formation and phase-separation behaviour of aqueous casein-alginate emulsions in the presence of strong polyelectrolyte. *Food Hydrocoll.* 25 350–360. 10.1016/j.foodhyd.2010.06.013

[B4] Bai S. Liu S. Zhang C. Xu W. Lu Q. Han H. (2013). Controllable transition of silk fibroin nanostructures: an insight into in vitro silk self-assembly process. *Acta Biomater.* 9 7806–7813. 10.1016/j.actbio.2013.04.033 23628774

[B5] Bhutani U. Laha A. Mitra K. Majumdar S. (2015). Sodium alginate and gelatin hydrogels: viscosity effect on hydrophobic drug release. *Mater. Lett.* 164 76–79. 10.1016/j.matlet.2015.10.114

[B6] Brachkova M. I. Marques P. Rocha J. Sepodes B. Duarte M. A. Pinto J. F. (2011). Alginate films containing lactobacillus plantarum as wound dressing for prevention of burn infection. *J. Hosp. Infect.* 79 375–377. 10.1016/j.jhin.2011.09.003 22000853

[B7] Bradford M. M. (1976). A rapid and sensitive method for the quantitation of microgram quantities of protein utilizing the principle of protein-dye binding. *Anal. Biochem.* 72 248–254. 10.1016/0003-2697(76)90527-3 942051

[B8] Calamak S. Erdoğdu C. Ozalp M. Ulubayram K. (2014). Silk fibroin based antibacterial bionanotextiles as wound dressing materials. *Mater. Sci. Eng. C* 43 11–20. 10.1016/j.msec.2014.07.001 25175182

[B9] de Moraes M. A. Beppu M. M. (2013). Biocomposite membranes of sodium alginate and silk fibroin fibers for biomedical applications. *J. Appl. Polym. Sci.* 130 3451–3457. 10.1002/app.39598

[B10] de Moraes M. A. Silva M. F. Weska R. F. Beppu M. M. (2014). Silk fibroin and sodium alginate blend: miscibility and physical characteristics. *Mater. Sci. Eng. C* 40 85–91. 10.1016/j.msec.2014.03.047 24857469

[B11] Derjaguin B. Landau L. D. (1941). Theory of the stability of strongly charged lyophobic sols and of the adhesion of strongly charged particles in solutions of electrolytes. *Acta Physicochimica U.R.S.S.* 14 633–662.

[B12] Doublier J. L. Garnier C. Renard D. Sanchez C. (2000). Protein-polysaccharide interactions. *Curr. Opin. Colloid Interface Sci.* 5 202–214. 10.1201/9780203755617

[B13] Draget K. I. Skjåk-Bræk G. Smidsrød O. (1997). Alginate based new materials. *Int. J. Biol. Macromol.* 21 47–55. 10.1016/S0141-8130(97)00040-8 9283015

[B14] Favre E. Nguyen Q. T. Clement R. Neel J. (1996). Application of flory-huggins theory to ternary polymer-solvents equilibria: a case study. *Eur. Polym. J.* 32 303–309. 10.1016/0014-3057(95)00146-8

[B15] Feng C. Song R. Sun G. Kong M. Bao Z. Li Y. (2014). Immobilization of coacervate microcapsules in multilayer sodium alginate beads for efficient oral anticancer drug delivery. *Biomacromolecules* 15 985–996. 10.1021/bm401890x 24502683

[B16] Grinberg V. Y. Tolstoguzov V. B. (1997). Thermodynamic incompatibility of proteins and polysaccharides in solutions. *Food Hydrocoll.* 11 145–158. 10.1016/S0268-005X(97)80022-7

[B17] Kim S. D. Chakravarti S. Tian J. Bell P. (2010). The phase behavior and the flory-huggins interaction parameter of blends containing amorphous poly(resorcinol phthalate-block-carbonate), poly(bisphenol-a carbonate) and poly(ethylene terephthalate). *Polymer* 51 2199–2206. 10.1016/j.polymer.2010.03.018

[B18] Kim U.-J. Park J. Kim H. J. Wada M. Kaplan D. L. (2005). Three-dimensional aqueous-derived biomaterial scaffolds from silk fibroin. *Biomaterials* 26 2775–2785. 10.1016/j.biomaterials.2004.07.044 15585282

[B19] Kim U. J. Park J. Li C. Jin H. J. Valluzzi R. Kaplan D. L. (2004). Structure and properties of silk hydrogels. *Biomacromolecules* 5 786–792. 10.1021/bm0345460 15132662

[B20] Koh L. D. Cheng Y. Teng C. P. Khin Y. W. Loh X. J. Tee S. Y. (2015). Structures, mechanical properties and applications of silk fibroin materials. *Prog. Polym. Sci.* 46 86–110. 10.1016/j.progpolymsci.2015.02.001

[B21] Kundu B. Rajkhowa R. Kundu S. C. Wang X. (2013). Silk fibroin biomaterials for tissue regenerations. *Adv. Drug Deliv. Rev.* 65 457–470. 10.1016/j.addr.2012.09.043 23137786

[B22] Leung V. Hartwell R. Elizei S. S. Yang H. Ghahary A. Ko F. (2014). Postelectrospinning modifications for alginate nanofiber-based wound dressings. *J. Biomed. Mater. Res. B. Appl. Biomater.* 102 508–515. 10.1002/jbm.b.33028 24155096

[B23] Li S. Li L. Guo C. Qin H. Yu X. (2017). A Promising Wound dressing material with excellent cytocompatibility and proangiogenesis action for wound healing: strontium loaded silk fibroin/sodium alginate (SF/SA) blend films. *Int. J. Biol. Macromol.* 104 969–978. 10.1016/j.ijbiomac.2017.07.020 28687393

[B24] Li X. Hua Y. Qiu A. Yang C. Cui S. (2008). Phase behavior and microstructure of preheated soy proteins and κ-carrageenan mixtures. *Food Hydrocoll.* 22 845–853. 10.1016/j.foodhyd.2007.04.008

[B25] Li X. Yan S. Qu J. Li M. Ye D. You R. (2018). Soft freezing-induced self-assembly of silk fibroin for tunable gelation. *Int. J. Biol. Macromol.* 117 691–695. 10.1016/j.ijbiomac.2018.05.223 29859277

[B26] Liang C. X. Hirabayashi K. (1992). Improvements of the physical properties of fibroin membranes with sodium alginate. *J. Appl. Polym. Sci.* 45 1937–1943.

[B27] Lopes L. M. De Moraes M. A. Beppu M. M. (2018). Study of phase separation in blends of silk fibroin and sodium alginate in solution and in solid state. *J. Polym. Res.* 25 198 10.1007/s10965-018-1594-3

[B28] Lu Q. Zhu H. Zhang C. Zhang F. Zhang B. Kaplan D. L. (2012). Silk self-assembly mechanisms and control from thermodynamics to kinetics. *Biomacromolecules.* 13 826–832. 10.1021/bm201731e 22320432 PMC3302850

[B29] Matsumoto A. Chen J. Collette A. L. Kim U. J. Altman G. H. Cebe P. (2006). Mechanisms of silk fibroin Sol-Gel transitions. *J. Phys. Chem. B* 110 21630–21638. 10.1021/jp056350v 17064118

[B30] Meng X. Tian F. Yang J. He C. N. Xing N. Li F. (2010). Chitosan and alginate polyelectrolyte complex membranes and their properties for wound dressing application. *J. Mater. Sci. Mater. Med.* 21 1751–1759. 10.1007/s10856-010-3996-6 20101440

[B31] Mession J. L. Assifaoui A. Lafarge C. Saurel R. Cayot P. (2012). Protein aggregation induced by phase separation in a pea proteins-sodium alginate-water ternary system. *Food Hydrocoll.* 28 333–343. 10.1016/j.foodhyd.2011.12.022

[B32] Ming J. Zuo B. (2014). A novel silk fibroin/sodium alginate hybrid scaffolds. *Polym. Eng. Sci.* 54 129–136. 10.1002/pen.23542

[B33] Mishra J. Tiwari S. K. Abolhasani M. M. Azimi S. Nayak G. C. (2017). “Fundamental of polymer blends and its thermodynamics,” in *Micro and Nano Fibrillar Composites (MFCs and NFCs) from Polymer Blends*, ed. Thomas S. (Amsterdam: Elsevier Ltd). 10.1016/B978-0-08-101991-7.00002-9

[B34] Nogueira G. M. De Moraes M. A. Rodas A. C. D. Higa O. Z. Beppu M. M. (2011). Hydrogels from silk fibroin metastable solution: formation and characterization from a biomaterial perspective. *Mater. Sci. Eng.* 31 997–1001. 10.1016/j.msec.2011.02.019

[B35] Pacek A. W. Ding P. Nienow A. W. Wedd M. (2000). Phase separation and drop size distributions in ‘Homogeneous’ Na-alginate / Na-caseinate mixtures. *Carbohydr. Polym.* 42 401–409.

[B36] Panouillé M. Larreta-Garde V. (2009). Gelation behaviour of gelatin and alginate mixtures. *Food Hydrocoll.* 23 1074–1080. 10.1016/j.foodhyd.2008.06.011

[B37] Popova E. Watanabe E. O. Pessôa Filho P. A. Maurer G. (2008). Phase equilibria for salt-induced lysozyme precipitation: effect of salt concentration and PH. *Chem. Eng. Process.* 47 1026–1033. 10.1016/j.cep.2007.02.005

[B38] Ribeiro M. De Moraes M. A. Beppu M. M. Monteiro F. J. Ferraz M. P. (2014). The role of dialysis and freezing on structural conformation, thermal properties and morphology of silk fibroin hydrogels. *Biomatter* 4:e28536. 10.4161/biom.28536 24646905 PMC4014454

[B39] Safronov A. P. Blyakhman F. A. Shklyar T. F. Terziyan T. V. Kostareva M. A. Tchikunov S. A. (2009). The influence of counterion type and temperature on flory-huggins binary interaction parameter in polyelectrolyte hydrogels. *Macromol. Chem. Phys.* 210 511–519. 10.1002/macp.200800495

[B40] Sarker B. Rompf J. Silva R. Lang N. Detsch R. Kaschta J. (2015). Alginate-based hydrogels with improved adhesive properties for cell encapsulation. *Int. J. Biol. Macromol.* 78 72–78. 10.1016/j.ijbiomac.2015.03.061 25847839

[B41] Schmid F. (2011). “Theory and simulation of multiphase polymer systems,” in *Handbook of Multiphase Polymer Systems*, eds Boudenne A. Ibos L. Candau Y. Thomas S. (New Jersey: John Wiley & Sons Ltd), 31–80. 10.1002/9781119972020.ch3

[B42] Shang S. Zhu L. Fan J. (2013). Intermolecular interactions between natural polysaccharides and silk fibroin protein. *Carbohydr. Polym.* 93 561–573. 10.1016/j.carbpol.2012.12.038 23499097

[B43] Sohn S. Strey H. H. Gido S. P. (2004). Phase behavior and hydration of silk fibroin. *Biomacromolecules* 5 751–757. 10.1021/bm0343693 15132657

[B44] Utracki L. A. Manias E. (2014). “Thermodynamics of polymer blends,” in *Polymer Blends Handbook*, eds Utracki L. A. Wilkie C. A. (Dordrecht: Springer), 517–675. 10.1007/978-94-007-6064-6

[B45] Wang Y. Fan S. Li Y. Niu C. Li X. Guo Y. (2020). Silk fibroin/sodium alginate composite porous materials with controllable degradation. *Int. J. Biol. Macromol.* 150 1314–1322. 10.1016/j.ijbiomac.2019.10.141 31747567

[B46] Wang Y. Wang X. Shi J. Zhu R. Zhang J. Zhang Z. (2016). A biomimetic silk fibroin/sodium alginate composite scaffold for soft tissue engineering. *Sci. Rep.* 6:39477. 10.1038/srep39477 27996001 PMC5172375

[B47] Wang Z. Yang H. Zhu Z. (2019). Study on the blends of silk fibroin and sodium alginate: hydrogen bond formation, structure and properties. *Polymer* 163 144–153. 10.1016/j.polymer.2019.01.004

[B48] Wenk E. Merkle H. P. Meinel L. (2011). Silk fibroin as a vehicle for drug delivery applications. *J. Control. Release* 150 128–141. 10.1016/j.jconrel.2010.11.007 21059377

[B49] Yang Y. Kwak H. W. Lee K. H. (2013). Effect of residual lithium ions on the structure and cytotoxicity of silk fibroin film. *Int. J. Indust. Entomol.* 27 265–270.

[B50] Yin Z. Wu F. Xing T. Yadavalli V. K. Kundu S. C. Lu S. (2017). A silk fibroin hydrogel with reversible sol-gel transition. *RSC Adv.* 7 24085–24096. 10.1039/c7ra02682j

[B51] Zhang H. Liu X. Yang M. Zhu L. (2015). Silk fibroin/sodium alginate composite nano-fibrous scaffold prepared through thermally induced phase-separation (TIPS) method for biomedical applications. *Mater. Sci. Eng. C* 55 8–13. 10.1016/j.msec.2015.05.052 26117733

[B52] Zhong J. Liu X. Wei D. Yan J. Wang P. Sun G. (2015). Effect of incubation temperature on the self-assembly of regenerated silk fibroin: a study using AFM. *Int. J. Biol. Macromol.* 76 195–202. 10.1016/j.ijbiomac.2015.02.045 25748848

